# Qualitative analysis of ward staff experiences during research of a novel suicide-prevention psychological therapy for psychiatric inpatients: Understanding the barriers and facilitators

**DOI:** 10.1371/journal.pone.0222482

**Published:** 2019-09-24

**Authors:** Yvonne F. Awenat, Sarah Peters, Patricia A. Gooding, Daniel Pratt, Charlotte Huggett, Kamelia Harris, Christopher J. Armitage, Gillian Haddock

**Affiliations:** 1 Division of Psychology and Mental Health, School of Health Sciences, Faculty of Biological, Medical and Health Sciences, Manchester, University of Manchester, United Kingdom; 2 Manchester Academic Health Science Centre, MAHSC, Manchester, United Kingdom; 3 Greater Manchester Mental Health NHS Foundation Trust, Manchester, United Kingdom; 4 Manchester Centre for Health Psychology, Division of Psychology and Mental Health, School of Health Sciences, Faculty of Biological, Medical and Health Sciences, University of Manchester, Manchester, United Kingdom; 5 Manchester University NHS Foundation Trust, Manchester, United Kingdom; ESIC Medical College & PGIMSR, INDIA

## Abstract

**Background:**

Suicide prevention is a global priority. Psychiatric hospitalization presents an opportunity to intervene positively with, for example, psychological therapies. However, evidenced-based suicide-prevention psychological treatments are rarely available on in-patient wards. Understanding staff engagement with research investigating suicide-prevention psychological treatments is crucial for their effective, efficacious, and pragmatic implementation. A pilot randomised control trial and feasibility study of Cognitive Behavioural Suicide Prevention therapy provided the opportunity for a qualitative investigation of staff experiences and views of a psychological intervention for people with suicidal experiences on psychiatric in-patient wards.

**Aims:**

To investigate staff acceptability of Cognitive Behavioural Suicide Prevention therapy for psychiatric inpatients based on their perceptions of their experiences during the conduct of a clinical trial.

**Method:**

Transcribed audio-recordings of qualitative interviews and a focus group (n = 19) of purposively sampled staff from eight psychiatric wards were analysed using inductive Thematic Analysis.

**Results:**

Facilitators and barriers were identified for: i) the conduct of the research, and, ii) the suicide-prevention intervention (Cognitive Behavioural Suicide Prevention therapy). Research-related barriers comprised communication difficulties between staff and researchers, and increased staff workload. Research-related facilitators included effective staff/researcher relationships, and alignment of the intervention with organisational goals. Suicide-prevention intervention-related barriers comprised staffs’ negative beliefs about suicide which impacted on their referral of inpatients to the clinical trial, and staff perceptions of insufficient information and unfulfilled expectations for involvement in the therapy. Facilitators included staff beliefs that the therapy was beneficial for inpatients, the service and their own clinical practice.

**Conclusions:**

Staff beliefs that ‘suicide-talk’ could precipitate suicidal behaviour resulted in covert gatekeeping and restricted referral of only inpatients judged as stable or likely to engage in therapy, which may not be those who could most benefit. Such threats to sample representativeness have implications for future therapy research design. The findings provide novel information for researchers and practitioners regarding the conduct of psychological treatment and research in psychiatric units.

## Introduction

Psychiatric inpatient fatalities account for between 3–5% of all suicide fatalities worldwide equating to 250 per 100,000 inpatient admissions [1]. Inpatient death by suicide is considered the most preventable of all suicides due to the 24 hour staff contact time with inpatients [2]. Despite this intensive contact time, 8% of UK patient suicides occur during psychiatric hospitalization, which can double to 16% within three months of discharge [3]. Hence, there is an urgent need for evidence-based treatments to prevent avoidable suicide deaths among psychiatric inpatients. This is of the highest priority for policy makers, service providers, clinicians, service users and carers both globally [4] and within the UK [5–7]. The primary function of mental health wards is purported to be the maintenance of inpatient safety by assessing and treating mental health problems [8]. The main criteria for admission is risk of harm to self or others [9]. Consequently, suicidal behaviour is common in psychiatric wards where self-harm rates of up to 68.8% of inpatients has been reported [10].

Psychological therapy has an established evidence base for suicide prevention [11–13], and Cognitive Behavioural Therapy (CBT) is recommended by the UK National Institute for Health and Care Excellence [14, 15] for the treatment of individuals who have suicidal thoughts and/or behaviours. However, provision of psychological treatment in inpatient settings is uncommon within the UK, where it is more likely to be offered post-discharge [16].

Within mental healthcare there is increasing awareness of the unsatisfactory gap between the existence of evidence-based treatments and their timely implementation into usual practice [17, 18]. Paradoxically, psychiatric treatments lacking rigorous evaluation are delivered [19], while treatments with demonstrated efficacy fail to be implemented [20]. Ingrained organisational cultures of resistance to change [21] that operate across all levels of mental health services [22] contribute to the difficulties of implementation of evidence-based treatments thereby preventing access by service users most in need [20].

It is recognized that avoidance of failure to achieve translation of evidence-based treatments into clinical practice requires consideration during the initial planning stages of early feasibility / acceptability research [23, 24]. This is particularly important regarding ‘complex interventions’ such as psychological therapy treatments [24].

One of the most important issues in translating the results of psychological treatment trials into clinical practice is that research trials may not reflect the real world environment of service users and clinicians. For example, research samples may be too homogenous [25]; manualised protocols used in trials may not be applicable to everyday settings [26]; and statistical significance may not equate to clinical significance [27].

Clinicians’ perceptions that research lacks clinical credibility can be a barrier to implementation of evidence-based treatments [28, 29] highlighting the importance of investigating staff acceptability during the development of new treatments [23, 24]. This is especially important within psychiatric wards where complex contextual challenges exist [30, 31]. Inpatient safety is of paramount importance to ward staff who are accountable for the care of large numbers of suicidal inpatients [9]. It is especially important to investigate ward staff views and perspectives regarding implementation of a clinical trial of suicide-prevention psychological therapy because, i) changes to staff’s usual working practices are necessary regarding their essential co-operation with participant recruitment procedures and facilitation of therapy delivery, and ii) evidence of adversarial attitudes and beliefs around caring for suicidal inpatients has been documented [30].

Mixed-method research designs incorporating qualitative and quantitative research [32] are recommended for the study of contextual factors [33–35]. We therefore conducted the INSITE study (Inpatient Suicide Intervention and Therapy Evaluation [36, 37] comprising a pilot Randomised Clinical Trial (RCT) with nested qualitative work-streams to evaluate the feasibility and acceptability of a suicide-prevention psychological treatment (Cognitive Behavioural Suicide Prevention therapy[38] [CBSP]) for suicidal inpatients.

### Brief summary of Cognitive Behavioural Suicide Prevention therapy

- CBSP[38] is a manualised treatment programme adapted by the authors for the inpatient setting [35]
- Up to 20 individual therapy sessions were offered over 6 months
- Initial therapy sessions were delivered on the ward once or twice weekly
- Session duration is typically up to 1 hour, but early sessions may be much shorter depending on inpatient’s psychological status
- Therapy usually continues weekly on the ward or in a community setting if the inpatient is discharged during the treatment programme
- CBSP is a recovery-based intervention aiming to develop / strengthen resilience whist also countering suicidal thoughts and behaviour
- The therapist aims to formulate an inpatient’s suicidal cognitions and behaviour and assist the inpatient to understand and make sense of these experiences
- CBSP treatment aims to change the cognitive processes underpinning suicide schema activation, maintenance and elaboration of suicidal thinking and behaviour using cognitive behavioural approaches.

The trial presented the opportunity to investigate staff perceptions of barriers and facilitators to the delivery of CBSP therapy for suicidal inpatients in a psychiatric inpatient setting. Ward staff, as key stakeholders, are likely to be highly influential in terms of constraining or facilitating treatment innovation [39]. Accordingly, the qualitative study reported here aimed to investigate and understand the views of ward staff during the conduct of the INSITE pilot RCT that was carried out in eight mental health wards within a large NHS mental health trust in the North West of England, UK [36]. There were two goals: First, to understand staffs’ perceptions of their experiences during the conduct of a clinical trial in the complex and challenging setting of acute psychiatric wards. Second, to investigate staff views regarding acceptability of the provision of a suicide-prevention psychological therapy for suicidal inpatients.

## Methods

Thematic analysis of individual, semi-structured qualitative interviews with staff working on psychiatric inpatient wards in the context of the INSITE pilot RCT[36].

### Epistemology and ontology

The epistemological stance underpinning this qualitative study was influenced by contextualism [40,41], which upholds the view that the social context within which people exist impacts symbiotically on how they understand and describe their experiences. This affiliated with our aim of understanding the experiences and views of psychiatric staff within the *particular context* of their role working with suicidal inpatient participants of the INSITE study. In tandem, our ontological stance is reflective of the belief in multiple realities (rather than a single truth) that are socially influenced and context dependent, thereby drawing on critical realist ontology [40,41].

### Analytic method

Thematic analysis [42] is a generic, theoretically-free, approach that is compatible with contextualism [42–44]. Thematic analysis[42] was selected for its: i) simplicity and flexibility in identifying patterns of meaning across the data corpus; ii) accessibility of use within mixed-method psychological research; and, iii) ability to produce results comprehensible to non-specialist audiences [43].

### Reflexivity

The authors uphold the principle that knowledge produced from qualitative investigations represents a co-produced endeavour within which researcher and participant both play an active part [45]. As most authors were working on the trial and some had developed the intervention, we understood that influences from our own backgrounds, beliefs and preconceptions could impact on biases affecting data generation and analysis [45–47]. Hence, in order to accurately portray participants’ own worldviews we engaged in reflexive self-scrutiny during analytic meetings [45–47] seeking to ‘bracket’ our own beliefs and preconceptions and adopt a stance of ‘empathic neutrality’ [48]

The first author, YA’s, professional background included over 30 years’ experience in senior general nursing positions within NHS hospitals prior to working in mental health research for over a decade. The other two qualitative interviewers (KM, CH), were both psychology graduates with experience of working in community mental health settings. The wider research team comprised senior psychology academics (SP, PG, CA) and clinical academics (GH, DP) with several years’ experience of conducting suicide prevention research. All but two authors (DP, CA) were female, including the authors who conducted the qualitative interviews (YA, KH, CH).

In presenting information at ward managers meetings and to staff who volunteered to participate in the qualitative research we outlined our roles and stated our aim of advancing evidence-based suicide prevention treatments by conducting research. Knowing that a participant’s relationship with the qualitative interviewer can impact on their responses[49] we delivered a standard introduction prior to every research interaction with staff stressing that the study aimed to investigate *feasibility and acceptability* rather than the efficacy of the intervention. We also stressed our wishes to hear their genuinely held views, and in particular to hear and understand about any difficulties encountered as a result of the study, as this would help us to design a high quality future definitive trial.

### Methodological rigour

We have aspired to conduct and to report a scientifically rigorous study, therefore to demonstrate methodological and reporting quality we have provided an inventory based on the Consolidated Criteria for Reporting Qualitative Research (COREQ)[49]. (S1 Appendix**)**

### Recruitment procedure

Members of staff were offered the choice to attend either a focus group or an individual interview. This was intended to, firstly, optimise uptake in recognition of staff difficulty in committing to time away from clinical duties [30] and, secondly, to facilitate personal choice preferences for individual or group interviews. This also enhanced methodological rigour as data triangulation is advocated to improve data quality[35]. Specifically, focus groups can encourage disclosure of potentially contentious views whilst individual interviews offer greater confidentiality [32, 50].

Purposive sampling [51] sought to recruit staff of varying grades and professions with experience of working in one of the eight the psychiatric wards across two hospital sites where the INSITE study was conducted[36]. Sampling continued until sufficient data were obtained to address the research aims[52] which was determined by consensus agreement of research team. Information about this qualitative study was presented by the first author (YA) at ward meetings, via posters on information boards, and through existing staff email systems. Those interested were provided with further verbal and written information and allowed a minimum of 24 hours to consider participation. Written informed consent was provided prior to participation. All researchers involved in recruitment and data collection were trained in qualitative research, research governance, ethics, and adhered to study standard operating procedures. Ethical approval was granted from the National Research Ethics Service Committee, Lancaster, North West England, UK (13/NW/0504).

### Participants

Of 31 staff expressing an interest, 19 people volunteered to participate. Reminder emails were sent to those who had initially expressed interest but no further replies were received. Of those who did participate, nine staff attended the focus group and ten were interviewed individually. Participants’ comprised three men and 16 women whose ages ranged from 22–56 years, their professional experience ranged from 2–27 years, and duration of employment in the host organization spanned from four months to 27 years. The sample reflected the full range of ward team roles, with 13 registered mental health nurses, two nursing assistants, three ward administrators and one psychiatrist. All participants worked in wards where the trial had been conducted and some had direct experience of working with inpatient participants recruited by the INSITE[36] study. At the time when the study was conducted there were no psychological therapists employed to work in the psychiatric wards.

### Data collection

A semi-structured interview schedule was developed with open questions [44] to elicit staff experiences, views and reflections of the conduct of the INSITE study (see S2 Appendix). Individual interviews were conducted by YA (n = 6) and KM (n = 4). The focus group was moderated by YA with assistance from KM and CH. All data collection occurred in private rooms in the participant’s workplace. Questions were designed to elicit information about staff expectations, views about provision of study information, and relationships and interactions with research staff. We also asked staff about their views of providing suicide-prevention psychological therapy for suicidal inpatients and invited views of any impact on inpatients who had received CBSP. Perceptions of how the research procedures and delivery of therapy had impacted on usual ward routines and workload were also sought. Staff were encouraged to suggest solutions to any challenges discussed and to freely introduce additional relevant topics that were not pre-defined in the interview schedule.

Field notes detailing relevant contextual issues were made to aide later analysis. Interviews and focus groups were digitally audio-recorded and transcribed verbatim during which identifying information was anonymised. Individual interviews ranged from 11–39 minutes duration (median = 18 minutes) and the focus group lasted 45 minutes. All audio-recordings were destroyed following transcription.

### Data analysis

Thematic analysis [42] was selected as a flexible, yet systematic method of analysing the data corpus of focus group and individual interviews. An inductive approach [42] to analysis was led by YA, with multiple coding to broaden the range of interpretative perspectives from SP, KH, and CH. All authors contributed to critical discussions to agree interpretation of the final themes. Analytic procedures followed Braun and Clarke’s [42–44] six-phase approach, commencing with several readings of the transcripts, prior to manual line-by-line scrutiny to identify relevant units of meaning as codes, which were then clustered together as tentative themes. There followed an iterative process of reviewing and restructuring the themes, culminating in the final thematic structure.

## Results

The analysis was organised into two discrete, yet interrelated areas depicting facilitators and barriers related to: i) research specific issues regarding the conduct of the RCT, and ii) staff views and beliefs regarding delivery of ward-based suicide-prevention psychological therapy for suicidal inpatients.

### Research related results

**Fig 1:**
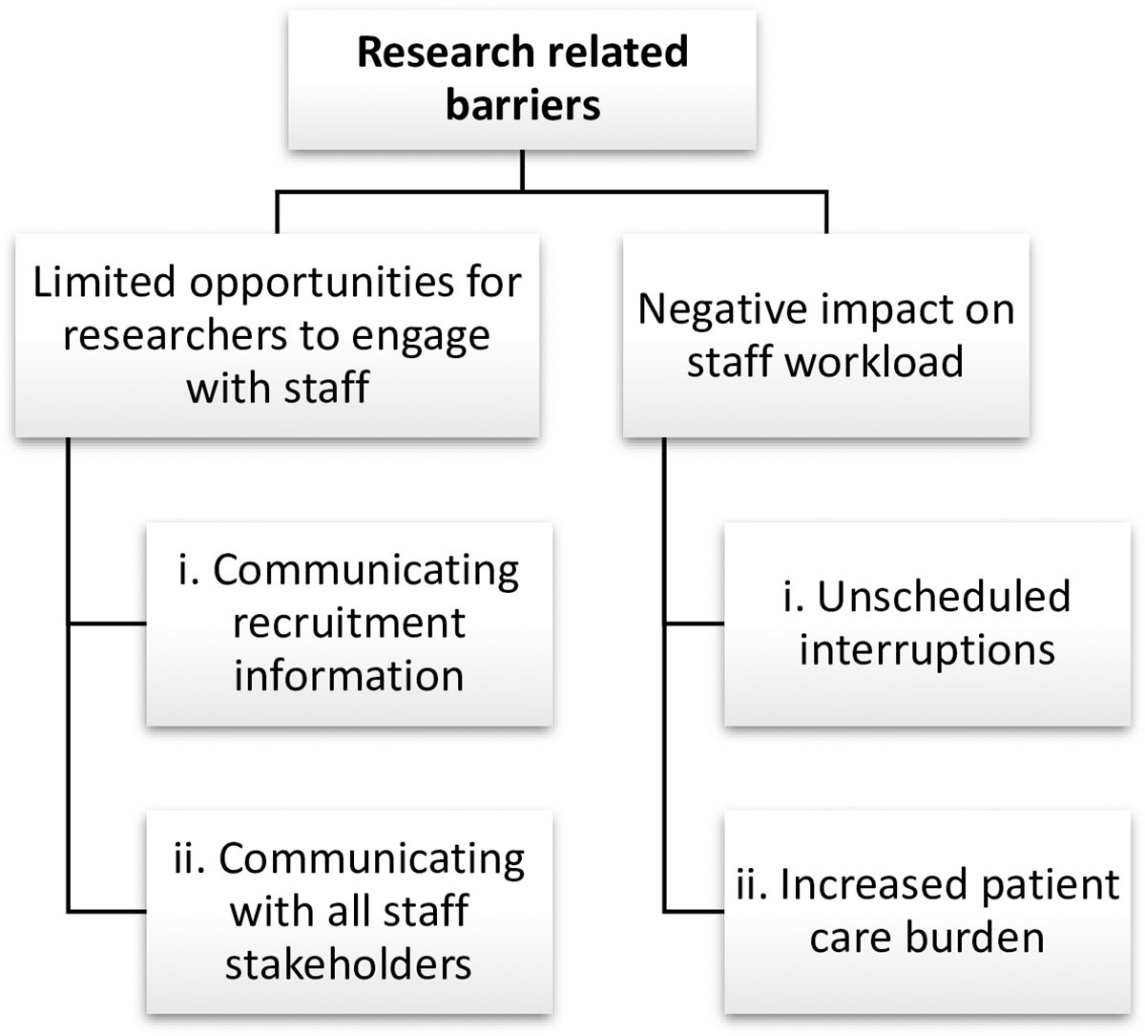
Research related barriers.

**Fig 2:**
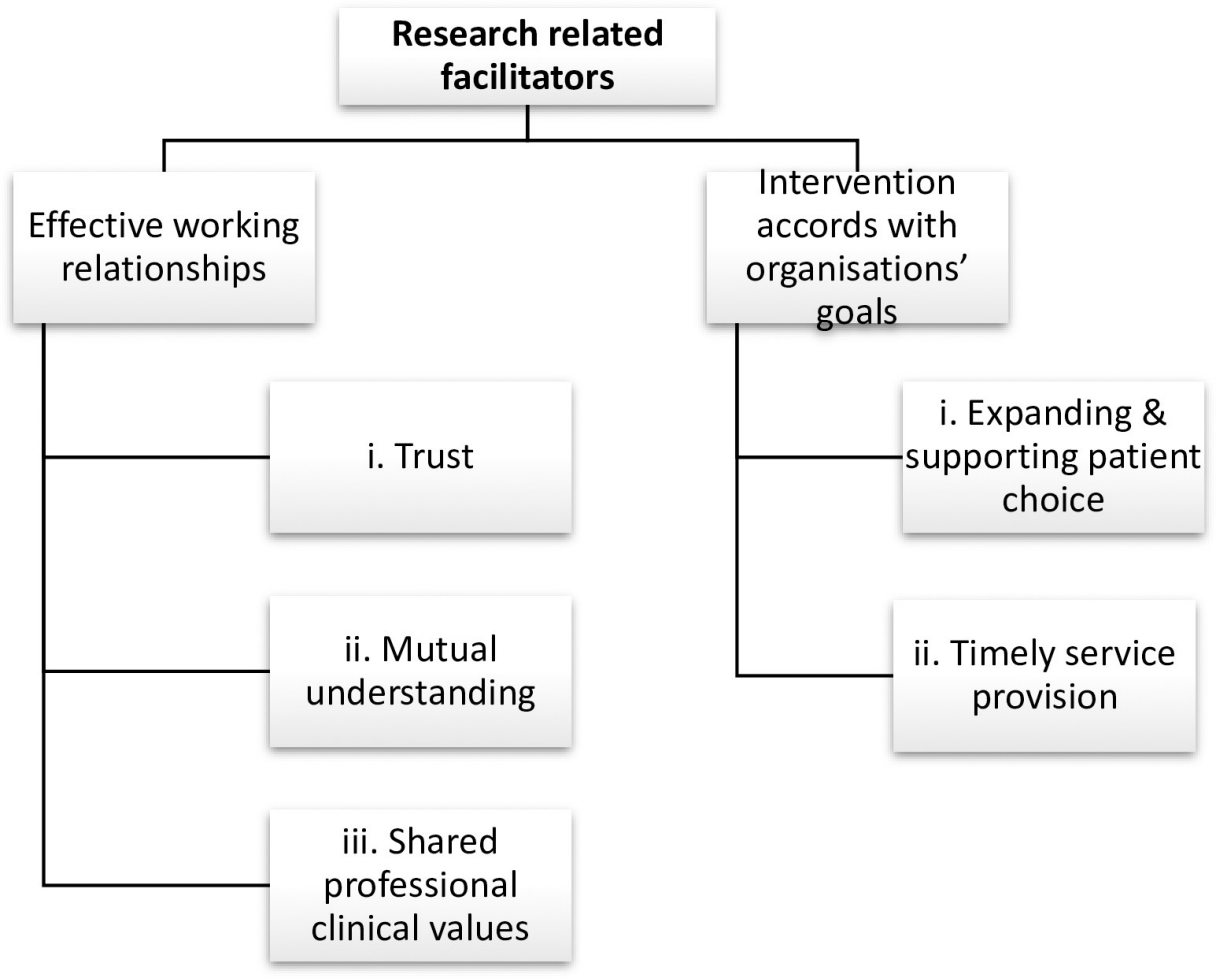
Research related facilitators.

Staff descriptions of their experiences of the research procedures revealed two main research related barriers (Fig 1) and two main research related facilitators (Fig 2) to the successful conduct of the RCT. Barriers comprised: (a) limited opportunities for researchers to engage with staff, and (b) negative impact on staff workload. Facilitators comprised (a) effective working relationships, and (b) research intervention accords with organization’s goals.

### Research related barriers

#### (a) Limited opportunities for researchers to engage with staff

Researchers were required to make contact with ward staff to provide information about the INSITE study and to agree methods of ongoing liaison with staff for the purpose of identifying potential participants for the trial and conducting research assessments.

#### (i) Communicating recruitment information

Recruitment of inpatients was dependant on referral by ward staff. It was, therefore, crucial to find acceptable ways of liaising with staff to provide study information. On commencement of the research, multiple methods of imparting introductory information to key opinion leaders at organizational and ward level included presentations at meetings, wall mounted poster information and staff internal email alerts. For some staff these methods were successful:-

“*I did see the information*. *I think it was good*, *it was concise*, *it wasn’t too much to read… It was quite straightforward and it*, *it kind of told you what the project was about properly*.” (009).

However, despite multi-faceted approaches, information failed to reach all staff with some reporting no knowledge of the research;

“*I’ve worked here two years*… *to be honest I’d never even heard of it until today*.*”* (FG038).

Communicating information to such large volumes of staff proved challenging as researchers encountered inconsistent availability of key permanent staff due to high staff turnover as illustrated by one participant;

“*Since this study started I’ve got completely different team*. *So I think eleven of staff members left*, *nine started and we were in a recruitment process*.*”* (011)

Forming relationships with key ward staff was further hampered by the unpredictability of their availability, which was determined by a system that rotated staff between day and night shifts. Another factor was the frequent use of external temporary agency nurses who were unfamiliar with strategic projects (such as research).

#### (ii) Communicating with all staff stakeholders

Particular difficulties were encountered in gaining access to ward team staff based elsewhere who only attended the ward periodically. It was, therefore, evident that certain staff groups, including psychiatrists, may have been overlooked;

“*some of the medical staff here weren’t really fully au fait with what [INSITE] was all about and*… *and they play just an integral part*.*”* (FG030)

Opportunities may also have been missed to engage with Health Care Assistants (HCAs) whose role involved greater direct contact with inpatients. As HCAs were more numerous than qualified nurses, they were potentially more available to provide assistance with recruitment, yet, it was suggested, they may have had little knowledge of the study;

“*the HCAs and the support staff that we’ve got*, *like [name] just said she doesn’t know anything about*, *but [name] might have a better relationship with the patients than I do*, *she might see them a lot more*, *and you know [name] primarily will be the one talking*.*”*(FG039)

#### (b) Negative impact on staff workload

Introduction of the INSITE trial onto the wards required certain organisational and behavioural changes to the usual work routines of staff that added to their already demanding workload.

#### (i) Unscheduled interruptions

Researchers made frequent visits to the wards to remind staff about the study, identify any eligible potential participants, and negotiate access to participants and interview rooms. Staff reflected on the difficulties experienced when receiving unannounced visits from researchers describing how this added to their stress and amplified the burden of their usual demands and competing priorities;

“*and a lot of the time*… *we never know what it’s gonna be like from one minute to the next*,.. *we lose out and the patient loses out then ‘cos you can’t really identify the most appropriate*, *‘cos like you could be in a restraint*, *you could be busy*, *you could be short [of staff] so it’s just like time*” (FG 032)

“*when you’re right in the middle of something and like got the phone going*, *you’re in the office on your own*, *and you’ve got*, *you know*, *people knocking at the door*… “(FG012)

#### (ii) Increased patient care burden

When staff were informed of an inpatients’ disclosure of suicidal intent during therapy or research interviews, it created additional demands on staff who had to provide more intensive levels of observation, support and record associated additional documentation;

“*And we was told about that [risk disclosure] and obviously* … *asked to start look at the patient*, *you know make sure the patient was safe and sometimes we had to write on [electronic record*].” (FG012)

Situations incurring additional workload were also described when inpatients’ became distressed following a therapy session, leaving staff believing that therapy was causing distress, leading them to feel more burdened and ill equipped to manage the ensuing outfall;

“*And then it’s left there and you’ve done a session and you’ve gone and then we’re left there while someone’s still feeling quite distressed*.*”* (FG017).

Such experiences influenced subsequent referral decisions as staff considered the potential for negative impact on individual inpatients and the consequent increase demands on staff;

“*One thing that you would worry about when you*, *when you’re nominating someone for the and the trial would be someone who would*, *talking about this could trigger maladaptive behaviour once the session had ended*, *and then we’d have a whole can of worms to put back in the can afterwards*. *So that’s a bit of a concern*.*”* (FG016)

A clear priority for staff was to maintain a calm ward environment and avoid unrest and conflict between staff and inpatients that would create additional workload. Some concerns were therefore expressed of the potential for resentment from inpatients’ who were not INSITE participants, and who might feel upset because they had been denied access to a treatment that others were receiving;

“*I think that would be difficult for some patients who would have thought*, *maybe if they thought that they were getting better treatment over them*, … *and*, *obviously*, *they are living together in quite close quarters for quite a bit so the last thing you want is a bit of resentment going on*, *on the ward*.. .. *they can be like that and compare themselves*, *‘Why do you get that and we don’t get that*?*’* … *So I think that could be… with the trial obviously*, *that could have been an issue”*. (005)

### Research related facilitators

#### (a) Effective working relationships

The negative impact of additional workload pressures was buffered by the perceptions of staff of having effective working relationships with researchers and therapists. This provided staff with a level of reassurance that mitigated concerns about maintaining the safety of suicidal inpatient participants.

#### (i) Trust

It was reported to be vital that ward staff could trust researchers and therapists to inform them of any heightened risk identified during research interviews or therapy. This was especially important as some staff were concerned that talking about suicide with researchers might increase inpatients’ suicide risk. Confidence that researchers and therapists would share any risk information with staff was viewed as an indicator of the quality of working relationships:

“*I think they worked really*, *really well*, … *if there was kind of information that we needed to know obviously about suicide risk they would hand that over to qualified staff so think the communication was really good*, *really good”* (009)

“*The research assistants*, *we developed quite a relationship with on our ward*, *and they were really good at providing feedback*, *if somebody had concerned them during the session*, *they were good at providing feedback about that and it was good to see that our ladies were quite clearly engaging with the researchers*.*”* (FG016)

#### (ii) Mutual understanding

Researchers and therapists visited the ward to attend meetings, discuss participant recruitment, conduct research assessments or see inpatients for therapy. Despite that staff sometimes perceived this to be an intrusion, they, nevertheless, tolerated this understanding that such interactions were necessary. The manner by which researchers and therapists demonstrated empathy and understanding of the pressures facing ward staff during occasions when they were unable to make time for liaison was appreciated and perceived to be influential in maintaining constructive relationships:

“*they (researchers) were always very*, *very accommodating*, *we’d be like*, *please can you just*, *do you mind even coming back tomorrow or come back in an hour*… *they were very understanding*, *yeah*, *‘cos they can see we’re snowed under*” (FG06).

**(iii) Shared professional clinical values.** Congruence of values formed the basis of a powerful bond that assisted the establishment of professional relationships between staff and researchers. For example, staff were supportive of the study as it addressed suicide prevention which was a high priority professional clinical value;

“*Well*, *the patient safety is always your number one priority*.*”* (FG016)

This shared value helped to maintain staff’s commitment to the study despite the difficulties encountered.

#### (b) Intervention accords with organization’s goals

Throughout interviews, staff alluded to their responsibility to uphold the NHS and organizational objectives of keeping patients safe, offering treatment choice and promoting recovery, all of which they recognised as embedded within the provision of CBSP therapy. Staff accounts portrayed positive attitudes towards the therapy that they believed would enhance suicidal inpatients’ treatment and improve their own professional practice, and therefore wished to support the study despite the demands this added to their workload.

#### (i) Expanding and supporting patient choice

Aside from any inherent benefits from receiving therapy, staff perceived a potential therapeutic value for some of their most vulnerable, often detained, inpatients from merely being *offered a choice* of treatment;

“*I think for our ladies [inpatients] it’s very empowering to be given the choice*, *because*, *a lot of our ladies come onto the ward and they’re very disempowered*.*”* (FG016)

**(ii) Timely service provision.** The opportunity for inpatients’ to access therapy during their suicidal crisis was perceived as timely and preferential to current practice that only offered post-discharge referral for therapy;

“*It’s like an in-reach service*, *so you’re actually providing a service for clients that they usually have been discharged to receive*, *so at the time when they most need it*, *when they’re inpatient*.” (FG016)

### Staff views and beliefs about Cognitive Behavioural Suicide Prevention therapy

Personal and professionally derived beliefs and attitudes were important determinants of how staff perceived the introduction of this novel suicide-prevention psychological therapy for suicidal inpatients under their care.

### Barriers to Cognitive Behavioural Suicide Prevention therapy

**Fig 3:**
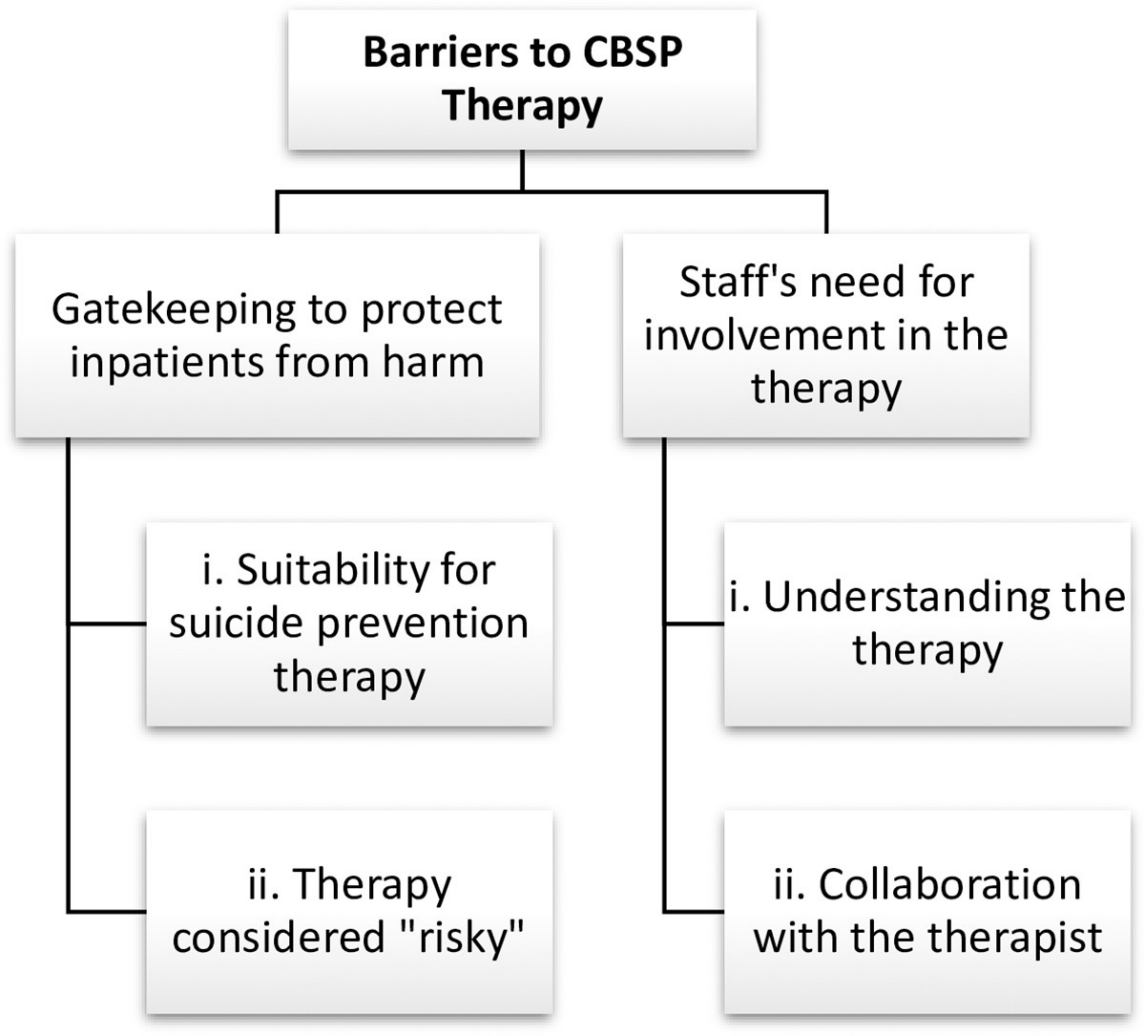
Barriers to Cognitive Behavioural Suicide Prevention therapy.

Two main barriers (see Fig 3) of ‘Gatekeeping to protect inpatients from harm’, and ‘Staff need for involvement in the therapy’ were evident. Introduction of CBSP challenged the pre-existing beliefs of staff about the appropriate treatment of suicidal inpatients, which were compounded by perceptions of insufficient knowledge of the therapy and lack of opportunity to collaborate with the therapist and to play an active part in the therapy process.

#### (a) Gatekeeping to protect inpatients from harm

Researchers sought to recruit inpatients who met the study inclusion criteria of being aged 18 years or older; having mental capacity to provide consent; and experience of suicidal thoughts or behaviours during the past three months. However, during the qualitative investigations it became apparent that staffs’ referral decisions were based on a range of other factors external to the study eligibility criteria.

#### (i) Suitability for suicide-prevention therapy

Staffs’ decisions regarding referral of inpatients to the study were influenced by their own views and judgements of who might benefit from therapy and who might become distressed or be de-stabilized by therapy rather than the study eligibility criteria. These included diagnosis, level of psychiatric symptom acuity and predictions of which inpatients would be interested and willing to engage in therapy;

“*You honestly just think*, *right who would be willing to sit and have a chat*? *‘cos you know some patients wouldn’t even entertain it*.*”* (FG032)

“*So if someone came in and was nowhere near ready to sit and engage*, *then we’d flag up as an appropriate referral*, *but say not just yet*.”(011)

Psychological therapy had not previously been available to inpatients in the wards where this study took place. Hence, beliefs that therapy was essentially a community treatment and was unsuitable for acutely unwell inpatients were evident:

“*it would be up to the discretion of the*, *like*, *that named nurse*, *whether they think that they are well enough or would benefit from participating*, *so the nurses would know*, *they’d think “oh they’ve been doing really well*, *they’re stable”*. *Or maybe people who are nearing discharge*.”(005)

**(ii) Therapy considered ‘risky’.** For some staff, the notion of encouraging suicidal inpatients’ to reflect on and discuss their suicidality during therapy, challenged their understanding and usual practices of working with suicidal inpatients. Staff whose practice aimed to avoid such discussions espoused the common lay belief that talking about suicide would precipitate suicidal behaviour;

“*in case you might say the wrong things… and that might*, *would trigger off a thought in them*.” (042).

When ‘suicide-talk’ was instigated by an inpatient, staffs’ usual practice comprised conversational techniques to distract and curtail such talk;

“*I’d just ask*, *‘How’s the weather*?*’ Or*, *you know*, *when a record comes on*, *‘Oh*, *what kind of music do you like*?*’ or just general chit-chat*, *which you would do with anybody*, *once they start chatting before you know it the hour’s gone*. *Another hour and I’ve kept them safe*.” (041)

Referral decisions were also influenced by staffs’ judgement of inpatients’ symptom acuity and the potential of becoming distressed and relapse following talk about sensitive matters including suicide;

“*I think*, *especially on my ward*, *it would be about how unwell they were*, *and it’s not necessarily how much they’ll engage*, *it’s about the risk to others*, *the risk to self*, *it’s about*, *if they’re*, *appropriate for discussions to talk about those things*, *… if they’re on like increased observations*, *or if they’re*, … *if there’s a certain date coming up that’s like a trigger point for them*. *if they*, … *we’ve got a few patients on ours that if they’re progressing but then reflecting back on certain incidents it can cause them to*,

” .. *to become quite unwell again*, *and then they’ll go right back to where they started*, *which takes quite a while to build them back up again*.*”* (FG039)

#### (b) Staffs’ need for involvement in the therapy

Staff were acutely aware of their responsibility to prevent suicide, and it was therefore important to them to be fully cognisant of factors that could impact on an individual inpatient’s risk status. The potential to miss a possible risk issue was of concern and staff would have liked more detailed information about the nature of the therapy and of individual inpatients’ progress during therapy.

#### (i) Understanding the therapy

Staff wanted to support access to CBSP onto the wards but had expected much more information about the structure, process and potential impact of the therapy which, they suggested, would have helped them to select suitable inpatients to refer to the study:

“*We didn’t really have any great knowledge of what it would be that patients would be doing and what benefits it might have*.” (035)

Staff were keen to learn about psychological approaches to suicide prevention including therapy and would have liked formal training which may have allayed some of their fears and uncertainties by increasing their knowledge and confidence in the therapy and research team:

“*Maybe have*, *like*, *a bit of a training course for all the staff*, *give us an insight into what you actually you do*” (041)

#### (ii) Collaboration with the therapist

Discussions revealed that staff would have liked greater collaboration with the therapist regarding inpatients’ progress in therapy, which they wished to understand and to be able to positively support the inpatient’s therapy journey. Staff portrayed a perception of carrying the burden of 24-hour responsibility for inpatients’ safety yet being denied important information perceived as necessary to fulfil this duty. They felt uncomfortable at not knowing about issues discussed during therapy, perceiving that more information and alerts from the therapist to particular inpatients’ idiosyncratic risks revealed during therapy would have enabled them to provide better support for therapy participants;

“*I think it obviously would be useful to get a kind of summary of what had been discussed*, *sort of the perspective of triggers to suicide risk and things like that*. *So we could understand a bit more*.” (009)

“*So it would be useful*, *as a team*, *especially if a named nurse to know just how well they’re engaging*… *it could be a trigger point for us as nursing staff*, *cos we have to manage their behaviour for the week”* (FG039)

Staff also expressed interest, and were curious about, the outcomes of inpatients who had been discharged and had continued their course of therapy in the community;

“W*hatever happened*? *Did they engage afterwards*? *Did they attend sessions*? *Did it go well*? *We just didn’t get that*, *there was a gap*… *that is the sort of stuff that will keep nurses motivated*, *‘cos it’s a lot of work to engage someone and then never find out what happened*.” (011)

### Facilitators to Cognitive Behavioural Suicide Prevention therapy

**Fig 4:**
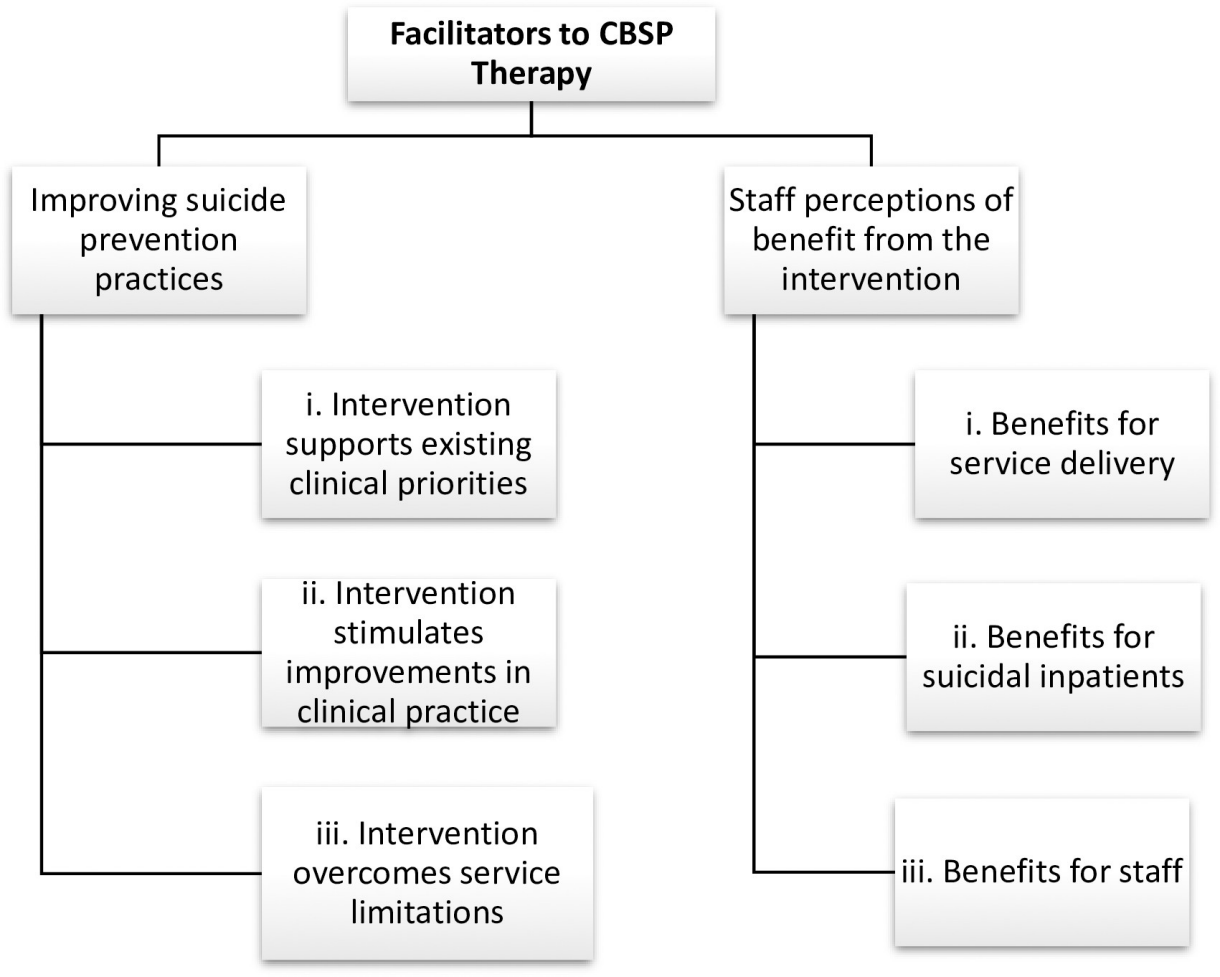
Facilitators to Cognitive Behavioural Suicide Prevention therapy.

Two main ‘Facilitators’ (see Fig 4) of: (a) ‘Improving suicide prevention practices’, and (b) ‘Staff perceptions of benefit from the intervention’ were identified. Although staff experienced conceptual and practical challenges resulting from introduction of access to CBSP in the context of a trial, they also described positive effects, which, by buffering the negative effects, facilitated its introduction.

**(a) Improving suicide prevention practices, (i) Intervention supports existing clinical priorities.** As patient safety and suicide prevention was of prime concern to staff, CBSP was viewed positively, particularly as it complemented other ongoing practice development projects. For example, the hospital was participating in a national quality improvement initiative named “Safewards”[53] which aimed to improve inpatients’ safety:

“*when it [the research] was starting we were starting the first time of implementing ‘Safewards’* … *so it fit in beautifully with the de-escalation modules and*, *mutual help*, *it fit along in*, *so yeah*, *people were receptive of it*.*”* (011)

#### (ii) Intervention stimulates improvements in clinical practice

Staff took the initiative to translate ideas gained from the study into usual practice to improve suicide prevention. For example, inpatient staff frequently conduct suicide risk assessments guided by a standard questionnaire yet some struggled to know how best to open conversations aimed at eliciting suicide risk and had found it helpful to use questions found on study flyers;

“*to have somebody say*, *when you’re asking about suicidal ideation*, *you could follow this*, *and you could include this in your risk assessment and that’s how we did it on our ward*, *so we incorporated the questions that had come through on the flyers*. *What it did do was help some of the newer and junior staff to use the questioning form*… *we used the template to get a more in-depth approach to suicidality*, *it’s shown in our risk assessments”* (011)

#### (iii) Intervention overcomes service limitations

Discussions revealed staff concerns that some suicidal inpatients were reluctant to trust them, resulting in an unwillingness to disclose their suicidal experiences, which staff attributed to inpatients’ knowledge of their legal powers of detention;

“*I mean there could well be a situation where I’m involved in sort of like enforcing treatment or doing something which might not all together be particularly welcomed*.*”* (FG033)

However, staff had noticed that the relationship was different between inpatients and their therapist who they were more willing to disclose suicidal ideation and acts to;

“*the patient may not be*, *feel comfortable to disclose things to us*, *they will disclose to the psychologist what was in them*, *and then we were able to intervene if we needed to support the patient*.” (034)

This provided reassurance to staff that suicidal inpatients would receive help from the therapist, who they were confidant would alert them to any increased suicide risk. In this situation staff appeared reassured to know that a ‘specialist’ was dealing with the ‘suicide specific’ issues enabling them to assume a more distal monitoring and support role;

“*It helps to give us some good insight into current thoughts of some of the patients who were happier to engage*, *maybe with the therapists than they were with the ward staff about their specific*, *specifics of how they were feeling*, … *so getting that feedback then*, *kind of let us know maybe which areas to further probe with the patient who let us think that everything was okay*… *and so I think everybody found it useful to have*, *kind of*, *objective opinion sometimes of what the current risks were because it can be very different how somebody presents themselves on a day-to-day basis and what they are really up to*.” (035)

#### (b) Staff perceptions of benefit from the intervention

Staff perceived several benefits of CBSP for suicidal inpatients, for the service, and for their own professional practice. Therapy was perceived to complement key existing professional practice and organisational ideals.

#### (i) Benefits for service delivery

Therapy for suicide prevention was recognised to fulfil an unmet need in current service provision for suicidal inpatients. Staff were aware of the limitations of current practice which primarily focussed on identifying imminent risk by completion of standard assessment forms, which, whilst quantifying static risk variables (e.g., previous attempts) failed to explore and address individual inpatient’s emotional experiences and psychological needs;

“*…they [risk assessment forms] tend to be*, *kind of*, *quite focused on immediate feelings of current mood*, *current*, *um*, *psychotic symptoms and the like*, *and so*, *although you can infer a lot from that*, *it doesn’t necessarily pull out exactly how somebody’s feeling*, *say about historical thoughts of self-harm*, *suicide and the like*, *which seems to have been something that came across better in the context of the therapy that the people were doing with the patients that came because they had the chance to*, *kind of*, *reflect on everything as a longer term measure*, *rather than what we a lot of the time are focused on*, *just the immediate risk*.*”* (035)

There was awareness of how the organisations’ need for documentation in itself contributed to objectification of the suicidal inpatient and legitimisation of a reductionist rather that a humanistic model of care;

“*I think we become very focused on risk and being mindful that we must assess that*, *document that*, *and respond to it in whatever way*, *and therefore it seems like it’s a bit mechanistic*, *its*, *it takes you away from entering the patient’s world in a way*. *That sounds a bit ironic*, *you’re talking to them*, *you’re speaking*, *asking their views… but*, *but*, *with*, *from the perspective of right*, *now*, *this is what we can do*, *we’re going to write this down*, *I’m not saying that we shouldn’t do those*, *we have to do those things*, *but it also*, *puts a bit of an unnatural barrier into stepping into those shoes for a minute*, *and trying to understand what that risk is relating to*.*”* (037)

Staff also anticipated that provision of CBSP could assist the achievement of key organisational targets for rapid discharge and avoidance of readmission;

“*I think it would help people to get discharged quicker*, *it would help people to not have repeat admissions all the time*.*”* (009)

#### (ii) Benefits for suicidal inpatients

Staff with direct experience of caring for inpatients who had received CBSP described several ways that they had observed therapy to provide benefit. Improvements in inpatients’ understanding of their suicidality along with enhanced personal agency to implement coping strategies learned in therapy were observed by staff to improve inpatients’ independence and ability to self-manage thereby reducing dependence on staff and services;

“*One patient who’d said she found it very helpful having the therapy*, *it had really*, *kind of*, *helped her understand a bit more about herself and what her risks were and how to manage her suicidal thoughts*… *I think its badly needed to be honest*, *I think it helps people become less dependent on services because they*, *kind of*, *learn to manage their own symptoms and their own feelings*, *and their own*, *kind of*, *suicidal ideas*, *or any*, *whatever it is they are working on*. *I think it encourages people to be a bit more independent and*, *it gives them a bit more autonomy*. *I think it’s really important*.*”* (009)

“*We noticed that they [inpatients] seemed to get a great deal of benefit from it*, *they were having an increased ability*, *maybe to reflect on their own behaviour*, *and so the*, *um*, *the study proved to be very useful in the end*, *as I say it wasn’t necessarily that before signing people up that we would have had a great deal of idea about what it was that was*, *kind of*, *involved but as it went on we could kind of see the benefit of it*.*”* (035)

#### (iii) Benefits for staff

Being aware of the potential of acclimatisation to suicide risk when continuously caring for large numbers of very acute and complex suicidal inpatients, staff welcomed the study as an opportunity to enhance and ‘refresh’ their practice;

“*when you work the wards a long time risk becomes part of your bread and butter- everyday management of somebody*, *so*, *to have an additional scheme that highlights and focuses in on suicidality and coping techniques as a direct line of working has been really helpful*” (011)

Working with suicidal inpatients was stressful but knowing that the therapist was working with the inpatient to offer focussed treatment for their suicidality helped to reduce staff anxiety and portrayed a sense of shared responsibility;

“*I feel a little bit apprehensive when someone comes that is suicidal*, *it’s something*, *a client group that I do get a bit nervous about*, *so I think it might help break down*, *sort of eliminate that nervousness*.” (041)

Collaboration with the therapist was valued by staff, who were able to gain a broader ‘psychological-formulation’ based understanding of an inpatient’s suicidal ideation and behaviour. This empowered staff to provide more focussed support and increased their confidence in their ability to keep suicidal inpatients safe;

“*It was useful for us to hear a different*, *sort of*, *side to what we were seeing*. *[Therapist] did build a plan with the patient and we worked through that and I put together a care plan from that information*… *I think it gave me something to use with this patient*, *to focus on and to*, *sort of reflect back on*, *if she’s struggling to say look at what you’ve done in your session*. *I think it helped*, *because to give us something to work on together cos I was her named nurse*” (040)

Staff reflected on how their practice might change if ward-based psychological therapy became available as a commissioned service, anticipating positive impact for their professional role development and more effective care delivery. Frustrations were expressed by staff who aspired to integrate psychologically informed approaches within their practice, but were inhibited by lack of organisational support structures including clinical supervision. Positive views were particularly evident regarding the potential of benefit for staff and inpatients of access to a ward-based psychological consultation facility that was currently unavailable;

“.. *support for the staff as well ‘cos there’s lots of staff that are trying to*, *that are trying to do psychological interventions*, *but they need the support of the clinical psychologist*.” (FG 033)

## Discussion

### Main findings

This is the first study to present a detailed analysis of psychiatric ward staff experiences and perspectives of delivery of a suicide-prevention psychological therapy for suicidal inpatients (CBSP). Our results advance new knowledge of value to psychological therapy researchers, practitioners and policy makers of: 1) the particular contextual factors that can inhibit or enable the successful conduct of psychological intervention research in acute psychiatric ward settings, and 2) how the role, beliefs and priorities of ward staff can impact to either facilitate or impede the provision of suicide-prevention psychological therapy for suicidal inpatients.

### Staff role and beliefs

Staff perceive working with suicidal inpatients as stressful, with some having experienced considerable distress and trauma following experiences of inpatient suicide [30]. It is, therefore, understandable that staff behaved risk-aversely in referring only those inpatients judged as stable and less likely to engage in suicidal behaviour to the INSITE trial. Reticence to initiate conversations about suicide may also have deterred some staff from providing initial information to suicidal inpatients about the INSITE study.

Initiatives requiring staff to change their behaviour, as required for implementation of a novel psychological therapy, will be affected by their expectations of the value of the novel intervention [54]. Therefore, it was important to examine staff views regarding the expected value of CBSP. We found mixed views. Some staff, whilst initially sceptical, became impressed following observed improvements in patients who were receiving CBSP. For others, the secondary effects from their interactions with the therapist led to positive expectations of the value of CBSP. Staff perceived that interactions with the therapist enabled them to acquire a greater understanding of the idiosyncratic psychological influences underpinning the suicidal behaviour of inpatients under their care. Such psychological formulation based insights thereby empowered staff to engage in more professionally rewarding and meaningful interactions with inpatients.

However, our results also revealed negative views of the expected value of CBSP as some ward staff feared that talking about suicide could ‘trigger’ suicidal behaviour. Similarly, staffs’ concerns of increased workload should inpatients experience post-therapy distress may also have contributed to negative expectations of the consequences of CBSP. Such views have previously been documented[32] and may explain staff gatekeeping resulting in restricted referral behaviour due to their concerns that CBSP may cause distress and provoke relapse. This is consistent with prior research purporting gatekeeping to be motivated by attempts to shield vulnerable patients from exposure to the perceived threats of a research intervention that clinicians lack confidence in [55–57].

Staff accounts also described perceptions of inadequate knowledge of the structure and process of CBSP, which may also have impacted on their ability to make informed judgements of its expected value. Potentially then, intuitive beliefs may have dominated resulting in reluctance to refer inpatients to the trial to avoid exposing vulnerable suicidal inpatients to an intervention which staff knew little about and believed could increase suicide risk.

Inpatient nursing activity is largely defined by pressures and events beyond the control of the staff [58] who, despite being charged with the serious responsibility of caring for suicidal inpatients, have limited control over ward life. Our research protocol required that staff initiate discussions with inpatients to provide information about INSITE and gain consent for researchers to meet them to give further information about the trial. However, situations characterised by organisationally defined priorities with inadequate staffing and unpredictable workloads would suggest challenges to staffs’ perceptions of control regarding their ability to find the time and the confidence to talk with inpatients about suicide. Similarly, staffs’ fears that inpatients could become distressed following therapy and subsequently require additional support may have triggered concerns related to staffing resources and skill deficits.

Staff imposition of unofficial additional eligibility criteria could potentially have threatened the external validity of results if this had continued undiscovered in a definitive trial. However, paradoxically a sample more closely reflective of those inpatients that clinicians’ would deem suitable to receive the intervention (i.e., a ‘real-world’ population) could more closely resonate with clinical practice [25]. This is because, had access to the therapy been delivered in the context of a commissioned service, rather than as a research intervention, referral of inpatients would occur at ward team meetings where broader discussion of service resource use and individual patient factors may be justified.

Consistent with our ‘contextualist’ [40] assumptions the results of the current study confirmed a commitment by staff to improving the treatment of suicidal inpatients, which was a fundamental facilitator for our study. An unexpected positive consequence was that some staff instigated changes to individual and ward level practices based on ideas arising from the study. For example, liaison with INSITE researchers and therapists, who were confident and comfortable to talk about suicide, provided a positive ‘modelling’ effect leading to staff emulating the more direct questioning style used by researchers to ask about suicidal thoughts or behaviour when conducting risk assessments.

### Impact of ward culture

It is important that researchers understand the real-world contextual challenges specific to conducting research in psychiatric wards which are typically chaotic and unpredictable environments [8–10, 58–60] Workforce instability is cited as a barrier to implementation of psychological approaches in psychiatric settings [61], and rapid staff turnover certainly inhibited our ability to form sustainable relationships with key clinicians.

Staff are accustomed to working in ‘fire-fighting’ mode to get themselves and the inpatients safely through their present shift [9, 10]. Whilst appreciating the complex personal, environmental and organisational challenges experienced by ward staff, such ‘short-termism’ presents challenges to conducting inpatient psychological therapy research and to improving the quality and efficacy of current treatments for suicidal inpatients.

Working in such stressful unstable conditions, staff understandably, prioritised ward management before the needs of researchers. However, as our results demonstrated, it was possible to negotiate and agree workable research procedures with staff, although ward preferences were idiosyncratic rather than global.

### Psychological therapy and inpatient settings

The difficulties of integrating psychological approaches into usual psychiatric ward practice has been described previously [59–63]. However, our results revealed surprisingly high levels of staff support for the introduction of CBSP, which could have been expected to challenge some traditionally held beliefs and practices. For example, ward purpose and culture prioritises rapid discharge achieved by containment and stabilisation of acute symptoms [8,9] rather than longer duration approaches (e.g., psychological therapy) to address the generic drivers of hospitalization. Distress is typically suppressed by sedative medication as opposed to psychological approaches enabling inpatients to discuss and find meaning from their experiences [25, 58]. Psychological therapy is not commonly provided in acute psychiatric wards, although such therapies may be suggested for post-discharge referral [16]. Hence, little is known about how staff would make decisions about referral to ward-based psychological therapy. In community settings the referral decisions of General Practitioners were found to be influenced by the clinician’s judgement of patient factors including the patient’s demand, motivation, insight, ‘psychological mindedness’, intelligence, education and the clinician’s prediction of the patient’s reliability of attending sessions [64]. Further research to investigate referral decision pathways for psychological therapy by inpatient staff is indicated.

Compatibility of a novel intervention with the organisation’s values and performance targets is recognised to be beneficial to staff uptake [21, 22, 29]. The compatibility of CBSP with local suicide prevention strategies may have been a contributory factor to staff engagement with the research, as despite additional workload pressures, staff generally welcomed and showed enthusiasm for CBSP.

Similarly, staff perceptions of positive collegial relationships with researchers and therapists appeared to be associated with the shared ‘burden’ of negotiating the challenges of preventing inpatient suicide. This may have exerted a buffering effect in countering some of the effects of increased staff workload. Staff accounts alluded to flaws in current models of suicidal inpatient treatment, valuing the involvement of the therapist, which provided some reassurance that therapy-arm suicidal inpatient participants’ were receiving a specific suicide prevention treatment not otherwise available.

### Real world clinical practice versus research practice

Staff discussed how their custodial role inhibited inpatients from disclosing suicidal thoughts or behaviours to them. This is corroborated by research of suicidal inpatients’ perspectives depicting their fears that disclosure of suicidality would prevent their desired discharge [65]. Hence, the combined impact of staff reluctance to engage in discussion about suicide and with suicidal inpatients’ reluctance to disclose suicidal thoughts to staff, suggests that staff are unlikely to be fully aware of which inpatients are suicidal. This highlights the need to consider participant recruitment procedures that do not rely solely on referral by ward staff. Under-recruitment to suicide intervention research is problematic [66] and when compounded by clinician gatekeeping [55–57] raises concerns about the external validity of some existing suicide intervention research where such factors may not have been considered.

Staff misinterpretation of their role by referring inpatient participants based on their own professional concerns rather than the research protocol defined criteria has serious implications for scientific rigour and is another example of the challenges of implementing standard RCT design for psychological therapy research in psychiatric ward settings [67]. We may have overestimated staff’s understanding of how the research processes necessary to maintain the parameters of a single-blind RCT of a novel treatment differ from those applicable when introducing a new commissioned service.

By striving to uphold the scientific principles of RCT design [68] that required controlling conditions so that outcomes could be confidently attributed to intervention effects, contact between the therapist and ward staff was limited to essential communication of disclosure of suicide risk. An unintended consequence was that staff desires for collaboration and active involvement in therapy patients’ treatment was thwarted. So, although staff desire to be actively involved in the therapy was a positive *facilitator*, paradoxically, the resultant unfulfilled expectations became a *barrier* to staff acceptance of the intervention. This may also have compounded staff perceptions of lack of control to fulfil their responsibility to maintain inpatients’ safe yet feeling excluded from a potentially ‘risky’ suicide-prevention treatment.

Similarly, whilst it was positive that staff were interested and requested training about psychological approaches to suicide prevention, this was withheld during the conduct of the trial to avoid confounding influences on trial outcomes. Arguably, additional training and any resulting changes in staff behaviour could confuse interpretation of whether RCT outcomes were attributable to CBSP, or to a ‘whole systems’ intervention comprising CBSP plus staff training. The latter may actually be a better approach for generating research, which, by more closely emulating real-world practice, is more likely to achieve translation into practice [67]. A potential solution to such challenges may be to consider alternative research designs for future ward-based psychological therapy research. In psychiatric research where standard RCT design was impractical, or threatened by contamination effects, pragmatic and standard cluster randomised controlled trials have been successfully implemented [69] including in inpatient settings [53].

### Strengths

This is the first study involving an in-depth investigation into how the role, perceptions and practices of psychiatric ward staff impact on the acceptability of introduction of a suicide-focused psychological treatment for suicidal inpatients. Our results, by illuminating the key barriers and facilitators, offer psychological interventionist researchers / clinicians specific guidance regarding the development and delivery of psychological interventions within psychiatric inpatient settings, which has hitherto been lacking. Flexibility within our research design by offering the choice of attendance at an individual interview or focus group facilitated good recruitment from ward staff whose clinical demands presented challenges to uptake. In addition to the pragmatic benefits, choice of interview mode also allowed for staffs’ idiosyncratic preferences of enhanced confidentiality within 1:1 interviews, or for the peer support and group cohesion found in focus groups which can facilitate revelation of contentious views [50].

### Limitations

At the time when this study was conducted the host psychiatric facility served populations from the geographical area with the highest suicide rate in UK [70], and the fourth most socio-economically deprived area in England UK [71]. Population and service complexity may suggest that the views and perspectives of staff in this study which was confined to one NHS mental health facility could differ from those in other organizations with lower concentrations of suicidal patients, more stable workforces and different contextual factors.

All three interviewers were female and interviewees mainly comprised female nurses, therefore the views expressed may be influenced by gender and professional orientations. However, nurses are arguably the most influential stakeholder group regarding the introduction and evaluation of ward-based innovations and the participant sample reflected the typical ward workforce. Psychiatrists, who hold particular status in seniority and authority within wards, were underrepresented, hence it would be important to ensure greater inclusion in future research. As staff self-selected it is possible that those who participated held more positive views than others not represented, therefore the barriers to introduction of CBSP may be underestimated.

A further limitation is that this study was conducted by the researchers who were conducting the trial which may have inhibited staff from divulging their genuine views. Similarly, it is possible that our affiliation with the trial may have biased our interpretations of the data. However, we did explicitly encourage staff to give feedback of negative experiences and to suggest improvements and this strategy was successful as staff did inform us about many barriers.

### Recommendations

In terms of staffs’ referral of inpatients to the RCT, and based on their views that they were not given sufficient information about CBSP, it would have been beneficial to invest greater resources in provision of information about the therapy to improve staffs’ knowledge and confidence in the intervention. The provision of information about the therapy, qualifications and skills of therapists, and also of the extensive safety protocols built around therapy delivery should be included in discussions as this may offer staff more confidence to refer in accordance with the study eligibility criteria. Further, an explicit negotiated protocol regarding sharing of risk-pertinent information between staff and researchers may be of mutual benefit. Discussions with ward staff should take a proactive stance to exploring potential fears about increasing suicidal inpatients’ risk by talking about suicide as this opens the opportunity to counter such concerns with research evidence [72].

As provision of psychological therapy is uncommon within inpatient settings little is known of how ward staff make decisions regarding referral of inpatients. This would therefore be an important area to study in the future.

In terms of research practice in psychiatric wards the potential for contamination effects and the challenges of recruitment strategies that rely solely on ward staff to refer participants indicates the need for careful choice of appropriate research methods and designs. It is also important to be aware that clinical staff may be unfamiliar with research practice. Therefore, provision of information should include explanations of the need to obtain a representative sample strictly in accordance with specified eligibility criteria.

Staff suggested some solutions to improve communication and liaison practices including adoption of existing approaches used by other visiting professionals comprising advance booking of regular meeting dates. Other suggestions included attendance at weekly multi-disciplinary ward meetings by researchers, and liaising with the inpatient’s ‘named-nurse’ rather than any nurse available. However, our experience indicated extensive variations of preferred approaches across wards, therefore further research is required to find effective methods of communicating with large numbers of constantly changing staff populations across several locations.

Staffs’ essential role within the INSITE trial required their willingness, motivation and ability to change their usual work behaviours to support the recruitment and care of research participants. This study did not set out to examine staff change behaviour from a behaviour change theory perspective, however on post-hoc reflection it appears that some findings may map onto concepts within theories such as the Theory of Planned Behaviour (TPB) [73, 74], which has previously been applied within suicide research [75,76].

The TPB [73] suggests the importance of an individual’s normative beliefs regarding the expectations of important others (i.e., their managers). In this respect it may have been beneficial if staff had experienced greater support and encouragement from their senior managers to make explicit the organization’s formal commitment to the study, including their investment of significant funds in support of the study. Potentially, this may have encouraged staff cooperation by legitimizing use of their time for research related activity. However, this may have had limited impact if not practically supported by provision of additional staffing resources. It may be helpful to consider a more detailed examination of behavioural change factors in future studies.

### Summary of recommendations for research investigating the delivery of therapy in psychiatric inpatient units

- Identify and meet with key opinion leaders within the hospital hierarchy to engage their support, ensure inclusion of all professional groups
- Provide information of research team members experience and expertise, and of the relevant ethics, research governance and safety protocols.
- Preliminary investigations to understand the prevailing contextual influences
- Be aware that ward activity, culture and staff views may differ even within the same hospital unit
- Identify staff expectations and agree plans to meet identified needs (e.g., information needs)
- Whenever possible involve staff in decisions regarding future plans
- Be mindful of creating procedures that cause additional staff workload, aim to utilize existing ward processes where possible
- Avoid assumptions of staff knowledge of research procedures, be prepared to explain reasons for eligibility criteria, randomisation etc
- Ascertain ward staff experience of psychological therapy treatment programmes for inpatients
- Pre-empt potential contentious issues by proactive discussion (e.g., misconceptions that talking about suicide is harmful; inpatients must be stable to receive psychological therapy)
- Provide information of the specific therapy model to be introduced and the role required of ward staff (referral mechanisms, level of involvement in therapy, potential support needs of therapy recipients, e.t.c.)
- Agree best ways of liaising with ward staff, including preferred times and days and times to avoid.
- Develop a protocol for two-way sharing of risk-pertinent information.
- Be prepared to provide multiple information sessions to ensure optimal staff coverage across 24 hour shift systems

## Conclusions

This study aimed to understand ward staffs’ perceptions of the barriers and facilitators to introduction of ward-based CBSP therapy in the context of a clinical trial. Our results illuminated the real-world contextual challenges that impact on: i) the conduct of psychological research in psychiatric wards, and ii) staff acceptability of delivery of ward-based CBSP. Conducting a pilot clinical trial in psychiatric wards was achievable although was hampered by staffs’ reluctance / inability to find time to engage with researchers. This may be reflective of the lack of a research culture, as despite NHS rhetoric purporting the importance of clinical research [77], staff viewed research as somewhat divorced from clinical practice. Overall, our findings indicated that despite concerns regarding potential workload challenges, and the philosophical opposition of some ward staff, the introduction of CBSP therapy was generally welcomed. The scale and depth of such highly impactful data gained from this qualitative study confirms the value of conducting acceptability / feasibility studies prior to definitive trials of novel interventions [24,32,34]. As researchers we cannot rectify the reality of the unpredictable, often chaotic nature of everyday life in psychiatric wards, however, the results of this qualitative study have provided us with knowledge of the modifiable challenges that can be pre-empted and addressed in future research.

The beliefs of some staff that talking about suicide is harmful reflect systemic failings fuelled by inadequate educational preparation of mental health staff and resource deficient service delivery models. Such organisationally driven ward practices neither value nor resource staff time to talk with suicidal inpatients. The prevailing organisational goals within psychiatric wards of stabilisation of acute distress and rapid discharge are neither clinically nor cost effective, as repetition of suicidality is common, often necessitating re-admission and / or more intensive community services. The escalation of post-discharge suicide rates and the displacement of numerical statistics, but not actual suicide deaths, from inpatient settings to community crisis-intervention services [78] mainly illuminates the difficulties of preventing suicide in community settings rather than the increased success of treating suicidal inpatients. Effective interventions are urgently needed and psychological treatments, which are currently underexploited, demonstrate potential warranting further research [79].

## Supporting information

S1 Appendix Consolidated Criteria for Reporting Qualitative studies (COREQ) inventory. (DOCX)

S2 Appendix Interview schedule. (DOCX)

## References

[1] Martelli C., Awad H., Hardy P. In-patient suicides: epidemiology and prevention. Encephale. 2010;36 Supp 2 83–91 10.1016/j.encep.2009.06.011 20513465

[2] Appleby L., Shaw J., Kapur N., Windfuhr K., Ashton A., Swinson N., et al Avoidable deaths: Five-year Report of the National Confidential Inquiry into Suicide and Homicide by People with Mental Illness (NCISH). University of Manchester, UK 2006.

[3] National Confidential Inquiry into Suicide and Safety in Mental Health. Annual Report: England, Northern Ireland, Scotland, Wales. 2018 University of Manchester UK.

[4] WHO. World Health Organisation. *Preventing Suicide*: *A global imperative*. Geneva WHO 2014: Available at: www.who.int/mental_health/suicide-prevention/world_report_2014/en/ Accessed 24/01/19

[5] Department of Health. DH. Preventing Suicide in England. A Cross-Government Outcomes Strategy to Save Lives. 2012 London, UK Department of Health / Government

[6] Department of Health. DH. The Five Year Forward View for Mental Health. 2016 Mental Health Taskforce London, UK.

[7] Large M., Chung D., Davidson M., Weiser M., Ryan C. In-patient suicide. 2017: *Journal of Psychiatry Open*. 3 102–105. 10.1192/bjpo.bp.116.004309 PMC5410408 28507768

[8] Bowers L., Simpson A., Alexander J., Hackney D., Nijman H., Grange A., Warren J. The nature and purpose of acute psychiatric wards: The tompkins acute ward study. *Journal of Mental Health*. 2009:14 (6).625–635

[9] Bowers L. Reasons for admission and their implications for the nature of acute inpatient psychiatric nursing. *Journal of Psychiatric and Mental Health Nursing*. 2005:12 (2) 231–236. 10.1111/j.1365-2850.2004.00825.x 15788042

[10] James K., Stewart D., Bowers L. Self-harm and attempted suicide within inpatient psychiatric services: A review of the literature. *International Journal of Mental Health Nursing*. 2012: 21 (1) 301–309. 22340085 10.1111/j.1447-0349.2011.00794.x

[11] Hawton K., Witt K., Taylor Salisbury T., Arensman E., Gunnell D., Hazell P., Townsend E. Van Heeringen K. et al Psychosocial interventions following self-harm in adults: a systematic review and meta-analysis. *The Lancet*. 2016:3(8) 740–750. 10.1016/S2215-0366(16)30070-0 27422028

[12] Winter D., Bradshaw S., Bunn F., Wellsted D. A systematic review of the literature on counselling and psychotherapy for the prevention of suicide: 1. Quantitative outcome and process studies. *Counselling and Psychotherapy Research* 2013:13(3) 164–183.

[13] Winter D., Bradshaw S., Bunn F., Wellsted D. A systematic review of the literature on counselling and psychotherapy for the prevention of suicide: 2. Qualitative studies. *Counselling and Psychotherapy Research*. 2014:14(1) 64–79.

[14] National Institute for Health & Care Excellence. (NICE). Self-harm: the short term physical and psychological management and secondary prevention of self-harm in primary and secondary care. NICE CG 16 2004: Available at www.nice.org.uk/guidance/CG16 accessed 10/04/19

[15] National Institute for Clinical Excellence (NICE). Self-harm: Longer term management. CG133 2011: Available at: www.nice.org.uk/guidance/CG133 accessed 04/03/19.

[16] Durrant C., Clarke I., Tolland A., Wilson H. Designing a CBT service for acute inpatient settings: A pilot evaluation study. Clinical Psychology and Psychotherapy. 2007: 14 117–125

[17] Munk-Jorgensen P., Blanner Kristiansen C., Uwawke R., Larsen J., Okkels N., Christiansen B., Hjorth P. The gap between available knowledge and its use in clinical psychiatry. Acta Psychiatrica Scandinavica. 2015:132 441–450 10.1111/acps.12512 26463889

[18] Proctor E., Landsverk J., Aarons G., Chambers D., Glisson C., Mittman B. Implementation research in mental health services: an emerging science with conceptual, methodological, and training challenges. Adm Policy Mental Health. 2009: 36 24–34 10.1007/s10488-008-0197-4 PMC3808121 19104929

[19] Gotham H. Diffusion of mental health and substance abuse treatments: Development, dissemination, and implementation. *Clinical Psychology*: *Science and Practice*. 2004:11(2) 160–176.

[20] Haddock G., Eisner E., Boone C., Davies G., Coogan C., Barrowclough C. An investigation of the implementation of NICE-recommended CBT interventions for people with schizophrenia. *Journal of Mental Health*. 2004: 23(4) 162–165. 10.3109/09638237.2013.869571 24433132

[21] Aarons G. Measuring provider attitudes toward adoption of evidence-based practice: Organizational context and individual differences. Child and Adolescent Psychiatric Clinics of North America.2005:14 255–271. 10.1016/j.chc.2004.04.008 15694785 PMC1564127

[22] Aarons G., Sawitzky A. Organizational culture and climate and mental health provider attitudes toward evidence-based practice. Psychological Services. 2006:3 *(*1*)* 61*–*72 10.1037/1541-1559.3.1.61 17183411 PMC1712666

[23] Cooksey D. A Review of UK Health Research Funding. 2006 Norwich HMSO. 10.1136/bmj.39059.444120.80 PMC1702444 17170394

[24] Medical Research Council. UK. MRC. (2008). *Developing and evaluation complex interventions*: *New guidance*. 2008: London MRC Available at: www.mrc.ac.uk/complexinterventionsguidance.

[25] Kennedy-Martin J., Curtis S., Faries D., Robinson S., Johnson J. A literature review on the representativeness of randomized controlled trial samples and implications for the external validity of trial results. Trials. 2015:16 495 10.1186/s13063-015-1023-4 26530985 PMC4632358

[26] Mullen A. Mental health nurses establishing psychosocial interventions within acute inpatient settings. *International Journal of Mental Health Nursing*. 2009:18(2) 83–90 10.1111/j.1447-0349.2008.00578.x 19290971

[27] Heneghan C., Goldacre B., Mahtani K. Why clinical trial outcomes fail to translate into benefits for patients. Trials. 2017:18 122 10.1186/s13063-017-1870-2 28288676 PMC5348914

[28] Retsas A., Barriers to using research evidence in nursing practice. *Journal of Advanced Nursing*. 2000:31(3) 599–606 10.1046/j.1365-2648.2000.01315.x 10718879

[29] Rycroft-Malone J., Harvey G., Seers K., Kitson A., McCormack B., Titchen A. An exploration of the factors that influence the implementation of evidence into practice. *Journal of Clinical Nursing*. 2004:13 913–924 10.1111/j.1365-2702.2004.01007.x 15533097

[30] Awenat Y., Peters S., Shaw Nunez E., Gooding P., Pratt D., Haddock G. Staff experiences and perceptions of working with in-patients who are suicidal: qualitative analysis. *British Journal of Psychiatry*. 2017: 10.1192/bjp.bp.116.191817 28642259 PMC5537568

[31] Laker C., Callard C., Flach C., Williams P., Sayer J., Wykes T. The challenge of change in acute mental health services: measuring staff perceptions of barriers to change and their relationship to job status and satisfaction using a new measure (VOCALISE). *Implementation Science*. 2014:9 (23) 10.1186/1748-5908-9-23 PMC4016533 24555496

[32] Tashakkori A., Teddlie C. *Mixed Methodology*. *Combining Qualitative and Quantitative Approaches*. 1998 London Sage

[33] Cochrane L., Olsen C., Murray S., Dupuis M., Tooman T., Hayes S. Gaps between knowing and doing: Understanding and assessing the barriers to optimal health care. *Journal of Continuing Education in the Health Professions*. 2007:27(2) 94–102 10.1002/chp.106 17576625

[34] Dixon T., Stein E., Ngak S., Srean C., Maly P., Sokunny M., Carrico A., Page K, Maher L. Qualitative research and implementation science: Informing the acceptability and implementation of a trial of a conditional cash transfer intervention designed to reduce drug and HIV. *Methodological Innovations*. 2016 10.1177/2059799115622751 30956811 PMC6448801

[35] Thurmond V. The point of triangulation. *Journal of Nursing Scholarship*. 2001: 33(3) 253–258. 11552552 10.1111/j.1547-5069.2001.00253.x

[36] Haddock G., Pratt D., Gooding P., Peters S., Emsley R., Evans E., Kelly J., Huggett C., Munroe A., Harris K., Davies L., Awenat Y. Randomised controlled trial of feasibility and acceptability of suicide prevention therapy in acute psychiatric wards. *British Journal of Psychiatry Open*. 2019 5(1) 10.1192/bjo.2018.85 PMC6381415 30762509

[37] Haddock, G., NIHR. 2014 A pilot study to investigate the feasibility and acceptability of a cognitive behavioural suicide prevention therapy for people in acute psychiatric wards. PB-PG-1111-26026. 2014. Final report to the National Institute for Health Research

[38] Tarrier N., Kelly J., Gooding P., Pratt D., Awenat Y., Maxwell J. *Cognitive Behavioural Prevention of Suicide in Psychosis*: *A treatment manual*. 2013 London Routledge.

[39] Rapp C., Etzel-Wise D., Mary D., Coffman M., Carlson L., Asher D., Callaghan J., Holter M. Barriers to evidence-based practice implementation: Results of a qualitative study. *Community Mental Health Journal*. 2010:46(2) 112–118. 10.1007/s10597-009-9238-z 19685185

[40] Madill A., Jordan A., Shirley C. Objectivity and reliability in qualitative analysis: Realist, contextualist and radical constructionist epistemologies. *British Journal of Psychology* 2000 91, 1–20. 10.1348/000712600161646 10717768

[41] Herbert J., Padovani F. Contextualism, psychological science, and the question of ontology. Journal of Contextual Behavioral Science. 2015: 4 225*–*230.

[42] Braun V., Clarke V. Using thematic analysis in psychology. Qualitative Research in Psychology. 2006: 3 77–101.

[43] Braun V. & Clarke V. Chapter 4. Thematic Analysis In American Psychological Association Handbook of Research Methods in Psychology. 2012 Vol 2 Research Designs, Cooper H. (Editor-in-Chief) American Psychological Association 10.1037/1362-004

[44] Braun V. & Clarke V. Successful Qualitative Research *A practical guide for beginners*. 2013 London Sage.

[45] Finlay L. Negotiating the swamp: the opportunity and challenge of reflexivity in research practice. *Qualitative Research*. 2 (2) 209–230.

[46] Mays N., Pope C. Qualitative research in healthcare. Assessing quality in qualitative research. *British Medical Journal*. 2000:320 50–52 10.1136/bmj.320.7226.50 10617534 PMC1117321

[47] Hellawell D. Inside-out: analysis of the insider-outsider concept as a heuristic device to develop reflexivity in students doing qualitative research. Teaching in Higher Education. 2006:11(4) 483–494

[48] Ritchie J., Lewis J., McNaughton Nicholls C., Ormston R. Qualitative Research Practice *A guide for social science students*. 2014:p22: (2^nd^ Edition) London Sage

[49] Tong A., Sainsbury P, Craig J. Consolidated criteria for reporting qualitative research (COREQ). *International Journal for Quality in Health Care*. 2007:19(6) 349–357 10.1093/intqhc/mzm042 17872937

[50] Lambert S., Loiselle C. Combining individual interviews and focus groups to enhance data richness. *Journal of Advanced Nursing*. 2008: 62(2) 228–237. 10.1111/j.1365-2648.2007.04559.x 18394035

[51] Marshall M. Sampling for qualitative research. Family Practice 1996 13(6) 522–525. 10.1093/fampra/13.6.522 9023528

[52] Dey I. Grounding grounded theory guidelines for qualitative inquiry. 1999 San Diego, Academic Press

[53] Bowers L., James K., Quirk A., Simpson A., SUGAR, Stewart D., Hodsell J. Reducing conflict and containment on acute psychiatric wards: The Safewards cluster randomised controlled trial. *International Journal of Nursing Studies*. 2015: 52(9) 1412–1422 10.1016/j.ijnurstu.2015.05.001 26166187 PMC4518134

[54] Armitage C., Norman P., Alganem S., Connor M. Expectations are more predictive of behaviour than behavioural intentions: evidence from two prospective studies. *Annals of Behavioral Medicine*. 2015:49(2) 239–246 10.1007/s12160-014-9653-4 25623893

[55] Borschmann R., Patterson S., Dilkushi P., Wilson D., Weaver T. Influences on recruitment to randomised controlled trials in mental health settings in England: a national cross-sectional survey of researchers working for the Mental Health Research Network. BMC Medical Research Methodology. 2014:14 23 10.1186/1471-2288-14-23 24533721 PMC3928923

[56] Bucci S., Butcher I., Hartley S., Neil S., Mulligan J., Haddock G. Barriers and facilitators to research in mental health services: Care coordinators’ expectations and experiences of referring to a psychosis research trial. Psychology and Psychotherapy. 2014: 88(3) 335–350 10.1111/papt.12042 25257960

[57] Mairs H. Lovell K., Keeley P. Clinician views of referring people with negative symptoms to outcome research. *International Journal of Mental Health Nursing*. 2012: 21(2) 138–144 10.1111/j.1447-0349.2011.00770.x 21951838

[58] Cleary M., Hunt G., Horsfall J., Deacon M. Ethnographic research into nursing in acute adult mental health units: A review. Issues in Mental Health Nursing 2011:32(7) 424–435 10.3109/01612840.2011.563339 21736465

[59] Holmes J. Acute wards: problems and solutions. Psychiatric Bulletin. 2002:26 383*–*385

[60] Flood C., Brennan G., Bowers L., Hamilton B., Lipong M., Oladapo P. Reflections on the process of change on acute psychiatric wards during the City Nurse project. *Journal of Psychiatric and Mental Health Nursing*. 2006:13 (3) 260–268 10.1111/j.1365-2850.2006.00932.x 16737492

[61] Woltmann E., Whitley R., McHugo G., Brunette M., Torrey W., Coots L., Lynde D., Drake R. The role of staff turnover in the implementation of evidence based practices in mental health care. *Psychiatric Services*. 2008:59(7) 732–737 10.1176/appi.ps.59.7.732 18586989

[62] Hurley J., & Rankin R. As mental health nursing roles expand, is education expanding mental health nurses? An emotionally intelligent view towards preparation for psychological therapies and relatedness. Nursing Inquiry.2008:15 (3) 199–205 10.1111/j.1440-1800.2008.00412.x 18786212

[63] McCann E., Bowers L. Training in cognitive behavioural interventions on acute psychiatric inpatient wards. *Journal of Psychiatric and Mental Health Nursing*. 2005:12(2) 215–222 10.1111/j.1365-2850.2004.00822.x 15788040

[64] Stavrou S., Cape J., Barker C. Decisions about referrals for psychological therapies: a matched-patient qualitative study. *British Journal of General Practice*. 2009:59(566) e289–e298 10.3399/bjgp09X454089 19761656 PMC2734375

[65] Awenat Y., Peters S., Gooding P., Pratt D., Shaw-Nunez E., Harris K., Haddock G. A qualitative analysis of suicidal inpatients views and expectations of psychological therapy to counter suicidal thoughts, acts and deaths. BMC Psychiatry. 2018: 10.1186/s12888-018-1921-6 30326878 PMC6192165

[66] Striman S., Brown G., Ghahramanlou-Holloway M., Fox A., Chohan M., Beck A. Participation bias among suicidal adults in a randomized controlled trial. Suicide and Life Threatening Behavior.2011:41(2) 203–209 10.1111/j.1943-278X.2010.00011.x 21470296 PMC3367501

[67] Hendricks Brown C., Curran G., Palinkas L., Aarons G., Wells K., Jones L., Collins L., Duan N., Mittman B., Wallace A., Tabac R., Ducharme L., Chambers D., Neta G., Wiley T., Landsverk J., Cheung K., Cruden G. An overview of research and evaluation designs for dissemination and implementation. Annual Review of Public Health. 2017:38 1–22 10.1146/annurev-publhealth-031816-044215 28384085 PMC5384265

[68] Sibbald B., Roland M. Understanding controlled trials: Why are randomised controlled trials important? *British Medical Journal*. 1998:316 201 10.1136/bmj.316.7126.201 9468688 PMC2665449

[69] Lovell K., Bee P., Brooks H., Cahoon P., Callaghan P., Carter L., Cree L., Davies L., Drake R., Fraser C., Gibbons C., Hinsliff-Smith K., Bower P. Embedding shared decision-making in the care of patients with severe and enduring mental health problems. The EQUIP pragmatic cluster randomised trial. PLOS One.2018: 10.1371/journal.pone.0201533 PMC6104914 30133461

[70] National Confidential Inquiry into Suicide and Homicide by People with Mental Illness (NCISH). Annual report. 2013 University of Manchester, Manchester. UK.

[71] UK Index of Deprivation (2015) http://www.gov.uk/government/statistics/english-indices-of-deprivation-2015 Accessed 10/11/18

[72] Dazzi T., Gribble R. Wessely S., Fear N. Does asking about suicide and related behaviours induce suicidal ideation. What is the evidence? Psychological Medicine. 2014 44(16) 3361–3363 10.1017/S0033291714001299 24998511

[73] Ajzen I. From intentions to actions: A theory of planned behaviour.p11-39, In Kuhl J. & Beckmann J., (Eds) Action Control: From Cognition to Behaviour. 1985 Berlin Springer-Verlag

[74] Armitage C. Connor M. Efficacy of the theory of planned behaviour; A meta-analytic review. *British Journal of Social Psychology*. 2001:40 471–499 11795063 10.1348/014466601164939

[75] O’Connor R., Armitage C. Theory of planned behaviour and parasuicide: An exploratory study. *Current Psychology*. 2003:22(3) 196–205

[76] Armitage C., Rahim W., Rowe R., O’Connor R. An exploratory trial of a simple, brief psychological intervention to reduce subsequent suicidal ideation and behaviour in patients admitted to hospital for self-harm. *The British Journal of Psychiatry*. 2016:208(5) 470–476 10.1192/bjp.bp.114.162495 26743808

[77] NHS England Research Plan. 2017 Leeds, UK. NHS England / Policy, Partnerships, & Innovation / Innovation and research Unit. Available at https://www.england.nhs.uk/wp-content/uploads/2017/04/nhse-research-plan.pdf accessed 19.06.19

[78] National Confidential Inquiry into Suicide and Homicide by People with Mental Illness. Making Mental Health Care Safer: Annual Report and 20 –year review (2016) University of Manchester, Manchester, UK.

[79] Hawton K. Pirkis J. Suicide is a complex problem that requires a range of prevention initiatives and methods of evaluation. *The British* Journal of Psychiatry (Editorial) 2017: 210 381–383 10.1192/bjp.bp.116.197459 28572430

