# Supplementary material for: Qualitative analysis of ward staff experiences during research of a novel suicide-prevention psychological therapy for psychiatric inpatients: Understanding the barriers and facilitators
Source: PLoS One. 2019 Sep 24;14(9):e0222482. doi: 10.1371/journal.pone.0222482 (PMC6759174; doi:10.1371/journal.pone.0222482)
Supplement: S1 Appendix — (DOCX) [file pone.0222482.s001.docx]

**S 1 Appendix Consolidated Criteria for Reporting Qualitative Studies (COREQ) inventory**

**Manuscript:** Qualitative analysis of ward staff experiences during research of a novel suicide-prevention psychological therapy for psychiatric inpatients: Understanding the barriers and facilitators.

This COREQ inventory follows the format suggested by Tong, Sainsbury and Craig (2007) and provides greater contextual detail of the stated requirements expected in a qualitative research publication.

**Domain 1: Research team & reflexivity**

***Personal characteristics***

1. *Interviewer / facilitator*

Of the nineteen Individual interviews, fifteen were conducted by the first author (YA), and four by co-author KM. The first author (YA) led moderation of the focus group assisted by CH and KM. (Stated in 2.7 Data Collection)

1. *Credentials*

All co-authors held appropriate academic and/or clinical qualifications to carry out this work and full details are stated in Title Page of the manuscript.

1. *Occupation*

All co-authors held either university academic positions or research staff positions with the study host NHS trust as indicated by organizational affiliations stated in Title Page of the manuscript.

1. *Gender*

This information is provided in section 2.3 Reflexivity. All staff who conducted the data collection were women (YA, KH, & CH).

1. *Experience and training*

The professional orientations and experience of the research team and all researchers directly involved in data collection (YA, KM, and CH) has been stated in Section 2.3. Reflexivity. All co-authors had training in qualitative research, ethics and research governance and adhered to the study standard operating procedures.

***Relationship with participants***

1. *Relationship established*

The prior relationship with staff participants is described in the Reflexivity section 2.3. All staff participants met with one of the interviewers / focus group facilitators in order to receive information about the qualitative study prior to their participation in an individual interview or the focus group.

1. *Participant knowledge of the interviewer*

Participants were informed of the role of the researchers as stated in section 2.3 paragraph.

1. *Interviewer characteristics*

Details of the co-authors who conducted the qualitative interviewers is stated in the Reflexivity section 2.3.

**Domain 2: Study design**

***Theoretical framework***

1. *Methodological orientation and theory*

Within the Methods section 2.0, the sub-section 2.1 ‘Epistemology and Ontology’ provides information of the underpinning theoretical framework for this study which was most closely aligned to epistemological contextualism and an ontological framework of critical realism. Similarly, selection of Thematic Analysis as the research method is described and justified in section 2.2 Analytic method. As the rich data resulting from the qualitative investigations was found to offer some important ‘messages’ relevant to the design of a further definitive trial the Theory of Planned Behaviour (TPB) was applied to some of the findings and this is outlined in the manuscript in the section 4.8, lines 841-855.

***Participant selection***

1. *Sampling*

Purposive sampling was selected to invite participation of individuals with the required experiences necessary to address the research aims and this is described in section 2.5.

1. *Method of approach*

Participants were approached by provision of information presented at ward meetings, staff information board posters, and by use of staff email communications as reported in section 2.5.

1. *Sample size*

Sample size was stated and referenced within the manuscript in section 2.5 as being determined by data sufficiency and was within established acceptable parameters.

1. *Non- participation*

The number of potentially eligible participants who declined to progress to participation was stated in the manuscript at section 2.5.

***Setting***

1. *Setting of data collection*

All data collection was carried out at the participant’s workplace which was a psychiatric unit and this is stated in the manuscript in section 2.7.

1. *Presence of non-participants*

Only the interviewers and staff participants were present during data collection and this is stated in the manuscript in section 2.7.

1. *Description of sample*

The demographic characteristic of the sample are described in the text of the manuscript section 2.6.

***Data collection***

1. *Interview guide*

The development, style and outline of content of the interview topic guide are described in the manuscript at section 2.7.

1. *Repeat interviews*

Each participant was only involved at one data collection event as stated in the manuscript at section 2.7.

1. *Audio / visual recording*

All data collection was audio-digitally recorded and this is stated in the manuscript at section 2.7.

1. *Field notes*

Notes concerning relevant issues were recorded by researchers during interviews and the focus group as stated in section 2.7.

1. *Duration*

Details of the range of durations of the individual interviews and the focus group are stated in the manuscript in section 2.7.

1. *Data saturation*

Cessation of sampling was directed by recognition that sufficient data was obtained in order to answer the aims of the research which is alluded to and referenced in the manuscript at section 2.5.

1. *Transcripts returned*

All transcripts were routinely quality checked for accuracy of documentation of content of the interview audio recording. As this qualitative study was conducted towards the end of the three year research funding period we did not have time nor staffing resources to offer return of transcripts.

**Domain 3: Analysis and findings**

***Data analysis***

1. *Number of data coders*

Multiple coding involved four researchers as detailed in the manuscript in section 2.8.

1. *Description of coding tree*

Coding followed Braun and Clarke’s suggested six-step model of data analysis as detailed in the manuscript in section 2.8

1. *Derivation of themes*

Coding followed Braun and Clarke’s suggested six-step model of data analysis as detailed in the manuscript in section 2.8.

1. *Software*

Software was not used for this study with analysis being carried out by traditional systematic manual procedures. Section 2.8

1. *Participant checking*

Participant checking was not carried out in this qualitative study

***Reporting***

1. *Quotations reported*

A range of quotations were reported throughout the Results in section 3 to illustrate themes and all were labelled with a participant ID number also indicating whether the data originated from an individual interview or from the focus group.

1. *Data and findings consistent*

In section 4 we have discussed and justified the consistency between the data and the interpretation of the findings

1. *Clarity of major themes*

The major themes are clearly stated in section 3.0 of the manuscript and for additional clarity are also presented as Figures, (Figures 1, 2, 3 & 4)

1. *Clarity of minor themes*

Minor themes (or sub-themes) are clearly situated and presented in the manuscript and for additional clarity are also presented as Figures, (Figures 1, 2, 3 & 4)
