# Supplementary material for: Qualitative analysis of ward staff experiences during research of a novel suicide-prevention psychological therapy for psychiatric inpatients: Understanding the barriers and facilitators
Source: PLoS One. 2019 Sep 24;14(9):e0222482. doi: 10.1371/journal.pone.0222482 (PMC6759174; doi:10.1371/journal.pone.0222482)
Supplement: S2 Appendix — (DOCX) [file pone.0222482.s002.docx]

**S2 Appendix Interview schedule**

**General views**

What are your overall views about the INSITE study?

**Prompt:** How did staff feel about the idea of introducing psychological therapy to help reduce suicidality in the ward?

**Explore:** Was it welcomed / viewed with concern / indifference?

**Views about the preparations for setting up the study**

What do you feel about the information given to ward staff before the study started?

**Prompts:** Was it enough, of the right amount, delivered in a helpful way?

How might we improve that if we were to start all over again?

**Views about integrating therapy into the ward routine**

What are your thoughts about the way researchers worked with ward staff during the study period?

**Prompts:** Any comments about where researchers were based / timings of visiting the ward?

What about relationships and communication between ward and research staff?

**Take suggestions for improvements**

What about your views of how therapy fitted into the busy ward routine?

**Prompts:** Did staff have any additional work demands as a result of the study taking place?

What worked well? Were there any benefits to staff?

What did not go so well?

How could it run better in the future?

**Views about impact of therapy on patients**

What do you think about how it affected patients?

**Prompts:** Were there any benefits for patients?

**Explore:** Can you describe these?

Were there any additional problems for patients receiving therapy?

What about those patients who were in the ‘Treatment as Usual group’ – were there any problems with that?

**Prompt:** Did they feel disappointed?

**Explore**: How could these difficulties be overcome in the future?

**Discharge planning of patients receiving therapy**

What about preparations for discharge when patients were still having their course of therapy?

**Prompt:** In what way did it impact on ward staff discharge planning procedures?

Invite comments on any other relevant issues by ward staff

**Views about the future**

Would you like to see psychological therapy more widely available in wards?

**If YES** - explore reasons & possible benefits and challenges

**If No** – explore why?

**Close.** Thank staff for attending & outline next steps
